# A National Dyadic Study of Spousal Education and Hypertension among Older Couples in the United States

**DOI:** 10.1093/geroni/igaf122.129

**Published:** 2025-12-31

**Authors:** Hui Liu, Wencheng Zhang, Juwen Wang

**Affiliations:** Purdue University, West Lafayette, Indiana, United States; Purdue University, West Lafayette, Indiana, United States; Purdue University, West Lafayette, Indiana, United States

## Abstract

Hypertension is a major public health concern, particularly for older adults. Guided by the linked lives perspective, this study examines the relationship between spousal education and hypertension risk among older couples. Using data from the National Social Life, Health & Aging Project (NSHAP) Round 3 (2015-2016), we analyzed 1,214 couple dyads aged 50 and older. Hypertension was assessed using both biological and self-reported measures. Spousal education was categorized by college degree attainment. Results from the Actor Partner Interdependence Model (APIM) suggest that a wife’s college degree was associated with lower odds of hypertension for both herself and her husband, but a husband’s college degree was not associated with the risk of hypertension for either spouse. Mediation analysis shows that husband’s health behaviors, but not household income, partly explained the association between a wife’s college degree and both spouses’ hypertension risk. These findings suggest that a wife’s education has a greater impact on a couple’s hypertension risk than a husband’s education, highlighting the need for interventions targeting couples with a lower-educated wife to promote cardiovascular health among older adults.

